# A comprehensive rat transcriptome built from large scale RNA-seq-based annotation

**DOI:** 10.1093/nar/gkaa638

**Published:** 2020-08-04

**Authors:** Xiangjun Ji, Peng Li, James C Fuscoe, Geng Chen, Wenzhong Xiao, Leming Shi, Baitang Ning, Zhichao Liu, Huixiao Hong, Jun Wu, Jinghua Liu, Lei Guo, David P Kreil, Paweł P Łabaj, Liping Zhong, Wenjun Bao, Yong Huang, Jian He, Yongxiang Zhao, Weida Tong, Tieliu Shi

**Affiliations:** Center for Bioinformatics and Computational Biology, Institute of Biomedical Sciences and School of Life Sciences, East China Normal University, Shanghai 200241, China; School of Basic Medical Sciences, Southern Medical University, Guangzhou, 510515, China; Center for Bioinformatics and Computational Biology, Institute of Biomedical Sciences and School of Life Sciences, East China Normal University, Shanghai 200241, China; Massachusetts General Hospital, Harvard Medical School, 51 Blossom St, Boston, MA 02114, USA; National Center for Toxicological Research, Food and Drug Administration, Jefferson, AR, 72079, USA; Center for Bioinformatics and Computational Biology, Institute of Biomedical Sciences and School of Life Sciences, East China Normal University, Shanghai 200241, China; Massachusetts General Hospital, Harvard Medical School, 51 Blossom St, Boston, MA 02114, USA; Center for Pharmacogenomics, School of Pharmacy, Fudan University, Shanghai, 200438, China; National Center for Toxicological Research, Food and Drug Administration, Jefferson, AR, 72079, USA; National Center for Toxicological Research, Food and Drug Administration, Jefferson, AR, 72079, USA; National Center for Toxicological Research, Food and Drug Administration, Jefferson, AR, 72079, USA; Center for Bioinformatics and Computational Biology, Institute of Biomedical Sciences and School of Life Sciences, East China Normal University, Shanghai 200241, China; School of Basic Medical Sciences, Southern Medical University, Guangzhou, 510515, China; National Center for Toxicological Research, Food and Drug Administration, Jefferson, AR, 72079, USA; Department of Biotechnology, Boku University Vienna, 1190 Muthgasse 18, Austria; Department of Biotechnology, Boku University Vienna, 1190 Muthgasse 18, Austria; Małopolska Centre of Biotechnology, Jagiellonian University, ul. Gronostajowa 7A, 30-387 Kraków, Poland; Biological Targeting Diagnosis and Therapy Research Center, Guangxi Medical University, Nanning 530021, China; SAS Institute Inc., Cary, NC, 27513, USA; Biological Targeting Diagnosis and Therapy Research Center, Guangxi Medical University, Nanning 530021, China; Biological Targeting Diagnosis and Therapy Research Center, Guangxi Medical University, Nanning 530021, China; Biological Targeting Diagnosis and Therapy Research Center, Guangxi Medical University, Nanning 530021, China; National Center for Toxicological Research, Food and Drug Administration, Jefferson, AR, 72079, USA; Center for Bioinformatics and Computational Biology, Institute of Biomedical Sciences and School of Life Sciences, East China Normal University, Shanghai 200241, China; Beijing Advanced Innovation Center for Big Data-Based Precision Medicine, Beihang University & Capital Medical University, Beijing, 100083, China

## Abstract

The rat is an important model organism in biomedical research for studying human disease mechanisms and treatments, but its annotated transcriptome is far from complete. We constructed a **R**at **T**ranscriptome **R**e-annotation named RTR using RNA-seq data from 320 samples in 11 different organs generated by the SEQC consortium. Totally, there are 52 807 genes and 114 152 transcripts in RTR. Transcribed regions and exons in RTR account for ∼42% and ∼6.5% of the genome, respectively. Of all 73 074 newly annotated transcripts in RTR, 34 213 were annotated as high confident coding transcripts and 24 728 as high confident long noncoding transcripts. Different tissues rather than different stages have a significant influence on the expression patterns of transcripts. We also found that 11 715 genes and 15 852 transcripts were expressed in all 11 tissues and that 849 house-keeping genes expressed different isoforms among tissues. This comprehensive transcriptome is freely available at http://www.unimd.org/rtr/. Our new rat transcriptome provides essential reference for genetics and gene expression studies in rat disease and toxicity models.

## INTRODUCTION

The rat (*Rattus norvegicus*) is utilized extensively as an animal model for studying human disease mechanisms and treatments. As an example, for toxicological studies, Gene Expression Omnibus (GEO) (1,2) currently hosts 1964 datasets using the rat model, the most among all species, followed by 1323 datasets for human. However, despite efforts to improve the annotation of the rat transcriptome based on cDNA and EST sequences, our knowledge on the transcriptome of rat is far from complete when compared with that of human and mouse.

RNA-seq technology enables an unbiased and in-depth analysis of the genome and transcriptome (3). Studies based on RNA-seq have revealed the complexity of the transcriptomes of eukaryotes (4,5) and have shown that many transcripts have escaped our observation. Examples include: much of the genome is transcribed, including the regions that were previously considered as junk DNAs; novel splice junctions were detected, demonstrating distinct splice sites and intricate patterns of alternatively spliced RNAs that may play important roles in the regulation and expression of the complex eukaryotic genome (5,6); most multi-exon genes are shown to have multiple alternatively spliced isoforms with different coding potentials.

Nowadays, RNA-seq provides direct RNA level resources for comprehensive transcriptome annotation with high sequencing depth and different types of samples and thus has been integrated into traditional annotation pipelines including Ensembl (7), ENCODE (8) and PASA (9,10). For example, the transcriptomes of zebrafish, human and fission yeast released by Ensembl, ENCODE (11), AceView (12) and Broad Institute (13), respectively, have been re-annotated with RNA-seq analysis.

Nevertheless, it is still challenging to construct a comprehensive transcriptome in the context of current RNA-seq data quality and computational methods. Generally, transcriptome reconstruction strategies with RNA-seq data fall into two categories, genome-independent and genome-guided. Genome-independent methods are preferred when the target organisms do not have a complete reference sequence, whereas genome-guided approaches, with their increased sensitivity in detecting transcripts, are used for annotating organisms with a reference genome (14,15). In addition, various pipelines and softwares have been published for transcriptome reconstruction, but they have limited compatibility (16), with notable inconsistencies of results. Hence, only a few software tools, data resources or analysis results based on large-scale RNA-seq technology are available for reusing in rat transcriptome analysis.

To generate a rat transcriptome with high resolution, we analyzed the rat BodyMap RNA-seq data generated by the SEQC consortium that consists of 320 deep-sequenced samples (>40 M reads per sample) from 11 different tissues (17,18). By carefully tuning the reconstruction pipeline, we obtained a relatively complete and reliable set of rat transcripts that was used to create a re-annotated transcriptome database and functional annotations comparable to that of the well-annotated mouse transcriptome. We confirmed the accuracy and reliability of RTR by comparing five other datasets derived from the same organs in RTR.

## MATERIALS AND METHODS

### Data source

The RNA-seq data were obtained from the rat RNA-seq transcriptomic BodyMap across 11 tissues, 4 developmental stages and both sexes generated by the SEQC consortium (*R*. *norvegicus*, strain: F344; GEO dataset: GSE53960) (17). Samples were prepared by Ribo-zero protocol. There are four biological replicates in each condition, i.e. samples sharing the same organ, the same development and the same sex. All of the datasets used for checking the precision of RTR were downloaded from the Sequence Read Archive (SRA) at NCBI (https://www.ncbi.nlm.nih.gov/sra) (Supplementary Tables S1 and 15) (19). The gene annotations of Rnor6 were obtained from the Ensembl database (version 97) (7), RGD (20), RefSeq (version 95) (21). The gene annotation of GRCh38/hg38 was obtained from the Ensembl database (version 97). The gene annotation of GRCm38/mm10 was obtained from the Ensembl database (version 97).

### RNA-seq read alignment and transcript assembly pipeline

The pipeline is depicted as a flowchart in Supplementary Figure S3. All RNA-seq short reads were first aligned to rat reference genome (rnor6) by HISAT2 (22). The aligned reads were assembled using two methods, Stringtie (23) and QuaPra (24), respectively. Post this initial assembly, we used Cuffcompare in Cufflinks suite to acquire common multi-exon transcripts in at least two biological replicates with FPKM > 1 in the same condition. If a novel multi-exon transcript found in only one replicate covers all the introns of another novel transcript which was found in at least two biological replicates with FPKM > 1 in the same strand, then the former transcript which obtained more exons was retained and the latter one was abandoned. A mono-exon transcript was defined as the one that the transcript should be detected in all four biological replicates with FPKM > 1 and then the leftmost 5′ end and rightmost 3′ end of these copies from four biological replicates are the boundary of the transcript. To obtain a tissue-specific transcriptome, the common transcripts in all four biological replicates from the same condition were merged using Stringtie-merge and only transcripts longer than 200 bp were retained. The common transcripts in all conditions were then merged using Stringtie-merge. The two transcriptomes derived by Stringtie and QuaPra were merged again. We then combined transcripts which were derived in the previous step with all the transcripts in Ensembl and RefSeq together into RTR. The performance of Stringtie and QuaPra are shown in Supplementary Figure S4, which indicates the consistency and complementarity between the two assemblers.

### Selection of newly annotated coding and noncoding transcripts with high confidence

In RTR, we predicted the transcript coding ability by incorporating three methods together: (i) CPAT (25), (ii) Pfam (26) and (iii) BLASTx (27). A newly annotated transcript in RTR was defined as a coding transcript with high confidence which simultaneously had a coding score greater than the cutoff in CPAT, had at least one Pfam domain and had high similarity with at least one known protein in all organisms in UniProtKB/Swiss-Prot where the similarity was estimated by BLASTx (*e*-value < 1e-5). Similarly, if a newly annotated transcript failed all of the three tests, it was then considered as a noncoding transcript with high confidence.

### Evidence for active regulation of transcriptional start sites

To conduct analysis of transcription start site (TSS) intervals, we downloaded the H3K4me3 ChIP-seq peak enrichment file (BED format) for the male liver at 9 weeks in rat (28). Intervals of ± 10 kb surrounding unique TSSs of transcripts in Ensembl and the newly assembled transcripts with high confidence were generated using the BEDTools slop tool (29). To control for expression, TSSs were filtered to remove transcripts not expressed in male liver at 6 weeks in rat (FPKM < 0.5). Base-wise peak coverage was generated using the BEDTools coverage function and summed per-base coverage histogram was normalized by dividing by the number of expressed TSSs.

### Identification of conserved splice junctions of RTR in human and mouse

Only transcripts with splice junctions were aligned. We used Needle in EMBOSS (30) to align the fragments close to novel junctions in coding transcripts with high confidence in rat to the fragments close to the junctions in the corresponding homologous genes in human and mouse, respectively (a region encompassing the nucleotides −6 to +6 relative to the splice junction was considered as ‘close’). Alignments were rejected in three cases: (i) there was more than one gap occurring close to the splice junction; (ii) when the junction in the transcript in human or mouse was not in the coding region, we used nucleotide alignment mode and matches with nucleotide identity <80% were rejected; (iii) when the junction in the transcript in human or mouse was in the coding region, we used protein alignment mode and matches with protein identity <80% or protein similarity <90% were rejected (physico-chemical properties of amino acid residues were used to assign similar residues: aliphatic, I, L, V; aromatic, F, Y, W, H; positive, H, K, R; negative, D, E; and tiny, A, C, T, S, G).

### Repetitive element analysis in RTR

RepeatMasker annotation for rat genome (rnor6 assembly) was downloaded from the UCSC database (31,32). intersectBed in BEDTools suite (33) was used to search the overlap between transposable elements (TEs) and exons. Anything that was not classified as a TE (such as low complexity, satellites and simple repeats) was removed from further analysis.

### Differential gene expression analysis and differential AS analysis

Pure read counts were extracted from the alignment files using featureCounts v2.0.1 (34). Genes which were considered to be expressed in at least one biological condition (i.e. a stage and tissue) (FPKM > 0.5) were retained. Subsequent differential gene expression analyses were performed using DESeq2 v1.20.0 guided by the RTR transcriptome (35).

rMATS v4.0.2 was used to screen differential AS events across different samples (36). The aligned data were run on rMATS for AS analysis. Then we calculated the differential AS events with the threshold of |ΔPercent spliced in (PSI)| > 0.05 and FDR < 0.05.

## RESULTS

### Re-annotated rat transcriptome

The characteristics of **R**at **T**ranscriptome **R**e-annotation (RTR) dataset and related known rat and mouse annotation resources are shown in Table 1, including the number of genes and transcripts, the size of transcribed region and the number and size of exons. By comparing the Ensembl transcript data between rat and mouse, it can be seen that the annotation of rat is much less complete than mouse in terms of both transcribed regions and exon regions. More than 55 000 genes and 140 000 transcripts are annotated in Mouse Ensembl release 97, while only ∼32 000 genes and 41 000 transcripts have been annotated in Rat Ensembl release 97. In contrast, our newly annotated rat transcriptome, RTR, identifies 52 807 genes and 114 152 transcripts, representing a substantial improvement over current rat transcriptome databases (Table 1, http://119.3.41.228:8888/static/RTR.gtf.gz).

**Table 1.** Summary of four rat transcriptome datasets (RTR, RGD, rat in RefSeq (version 95), Rat Ensembl release (version 97)) and Mouse Ensembl release (version 97)

	RTR	RGD	Rat in RefSeq	Rat Ensembl v97	Mouse Ensembl v97
Total number(G/T)	52 807/114 152	41 293/71 746	17 347/19 005	32 883/41 078	55 573/142 333
Transcribed region (bases)	1 210 629 410	1 146 889 108	745 274 636	866 345 921	1 166 497 934
Transcribed region ratio	0.422	0.400	0.260	0.302	0.427
Exon number	383 460	294 311	166 150	240 383	413 177
Exon region (bases)	189 460 026	93 815 592	39 838 763	56 335 223	116 671 536
Exon region ratio	0.066	0.032	0.014	0.020	0.043

*G* genes; *T* transcripts.

By incorporating the publicly available data resources (Ensembl (7), RefSeq (21)) with our RNA-seq data, we found that ∼42% of genomic regions were transcribed and ∼6.5% of genomic regions were identified as exon regions in the resulting RTR transcriptome. Although the length of transcribed regions in RTR is only slightly longer than that in RGD (https://rgd.mcw.edu/) which previously was the most comprehensive rat transcriptome (20), the sum of the identified exon region bases in RTR is twice as large as that in RGD. Additionally, the number of rat transcripts in the re-annotated transcriptome is 114 152, which is 2.78 times as large as that in Rat Ensembl (41 078) and comparable to that of the latest mouse annotations.

To confirm the authenticity of RTR, we checked the precision of multi-exon transcripts in five other datasets downloaded from the Sequence Read Archive (SRA) (https://www.ncbi.nlm.nih.gov/sra) (19). The five selected datasets correspond to organs overlapping those of the 320 samples in the SEQC consortium. We defined precision as the percentage of predicted transcripts in the five datasets that matched the transcripts in the corresponding tissues in RTR, respectively. The precision in each dataset is higher than 97% (Supplementary Table S1). There are 4638 transcripts longer than 200 bp in known databases (Ensembl, RefSeq, RGD) that were not found in the 320 samples in the SEQC consortium. Intriguingly, function analysis for these uncovered annotated transcripts implied that 856 of these transcripts are involved in the biological process of olfactory receptor using a hypergeometric test (Bonferroni-adjusted, *P*-value < 0.05) with data in Gene Ontology (37). Most of the 4638 transcripts were also not found in the five other datasets (Supplementary Table S1).

### More detected novel junction sites

Similar to the exome level data, 266 182 junctions were detected in this BodyMap data, which was over 1.33 times more than found in Ensembl. Unexpectedly, the junctions detected in the 320 samples were quite differently distributed than in other annotation databases (RGD, Ensembl and RefSeq). While splice junctions detected in this BodyMap data identified 94.4 and 93.3% of splice junctions in Ensembl and RefSeq, respectively, only 81.7% of junctions in RGD were identified, suggesting a higher proportion of unique splice junction candidates in the RGD database.

We also investigated the splice patterns of novel junctions. Splice junctions were generally flanked by a canonical GT-AG sequence and 97.18% of splice junctions in the RTR database showed this signature. In other words, the splicing sites in our identified novel junctions showed more canonical splicing patterns than the existing Ensembl database (93.3%).

### More identified alternative isoforms and splicing events

Analysis of the BodyMap data revealed a much greater extent of alternative transcripts than previous annotations. There are 73 074 newly annotated transcripts in RTR, 48 013 transcripts of which belong to 14 185 genes in Ensembl. As a result, the ratio of transcripts belonging to genes in Ensembl to the genes themselves increases from the original 1.30 to 2.71 in RTR. There are 19 924 newly annotated genes in RTR, and the ratio of transcripts in newly annotated genes to the newly annotated genes themselves is 1.26, which is comparable to the one in the known annotation in Ensembl (1.25).

**Figure 1.**
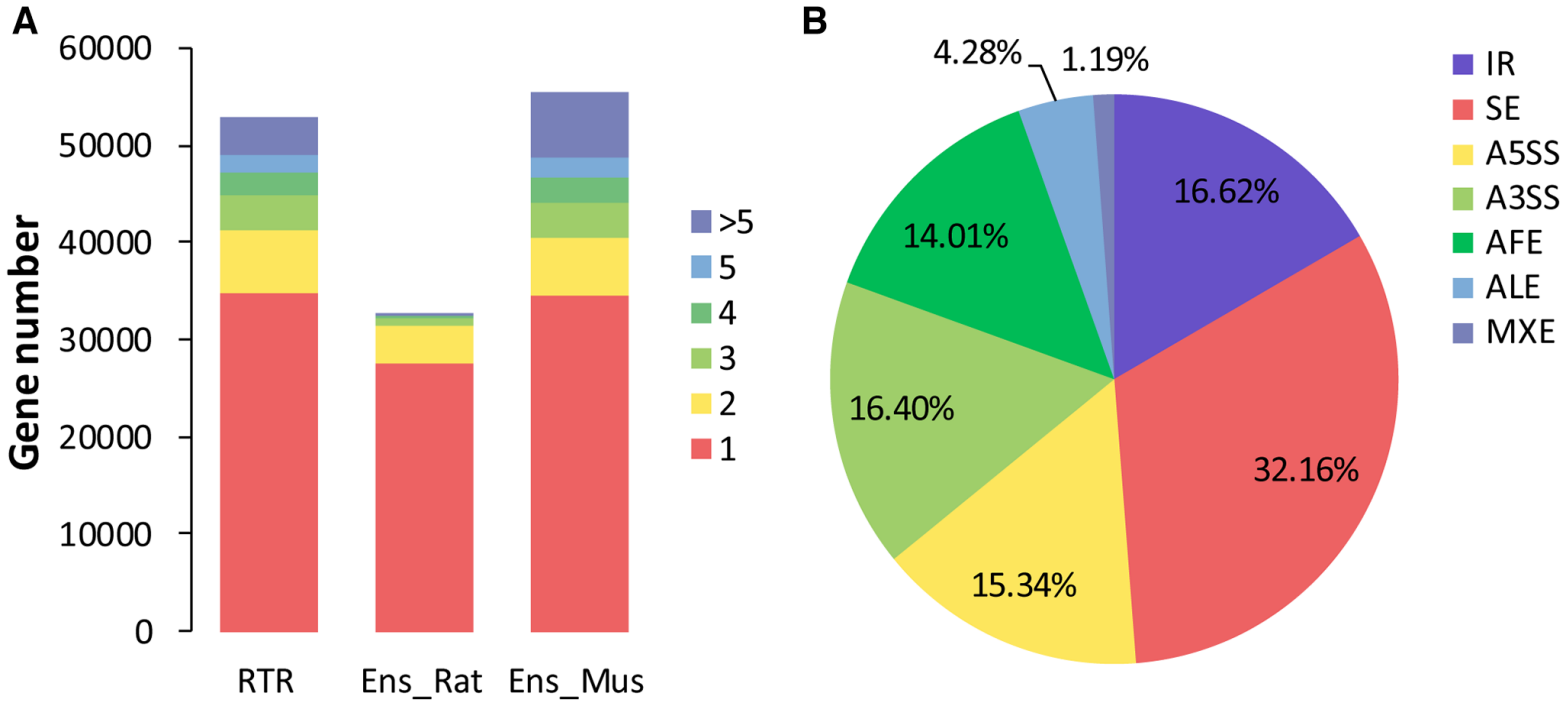
(**A**) The number of genes with different isoforms. Each block in a bar corresponds to the number of genes with multiple isoforms. Three transcriptomes were compared, from left to right: RTR, Rat Ensembl release and Mouse Ensembl release. (**B**) The distribution of AS events in RTR.

As shown in Figure 1A, 34.3% of rat genes in RTR have more than one isoform (multi-isoform genes), which is at an equivalent level to that of Mouse Ensembl release (version 97), while in Ensembl only 15.4% of rat genes have more than one isoform. In addition, rat multi-isoform genes in RTR have 4.39 isoforms on average, which is at an equivalent level to that of the mouse annotation of Ensembl (4.45), while in Ensembl rat multi-isoform genes have only 2.41 isoforms in average. Furthermore, the percent of rat genes with at least three isoforms in RTR (22.1%) is over six times as large as the one in Rat Ensembl release (version 97) (3.5%).

We further used ASTALAVISTA (38) to characterize these alternative splicing (AS) events into seven different categories: intron retention (IR), skipped exon (SE), alternative 5′ donor site (A5SS), alternative 3′ acceptor site (A3SS), alternative first exon, alternative last exon and mutually exclusive exons (MXE). The distribution of different AS events is similar between RTR (Figure 1B) and Ensembl (Supplementary Figure S1) with SE, the dominant mode of AS (39), being more prevalent in RTR than Ensembl. Retained intron splicing events accounted for only 16.62% of all AS events in RTR which suggests that RTR is not likely affected by the incomplete splicing of pre-mRNA.

Splicing factors (SFs), which are RNA binding proteins that play key roles in AS regulation, often drive widespread differences in AS patterns across different tissues through tissue- and cell-type-specific expression (40). Therefore, we further explored those SFs in rat to understand the mechanisms of various splicing events. By homology mapping to human SFs (41), we identified 84 SF genes expressed in rat (Supplementary Table S2). Interestingly, Esrp2 shows higher expression levels in liver, lung and kidney while it has very low expression levels in thymus and uterus; Elavl2 shows obvious tissue specificity, which is highly expressed in brain at all development stages while having very low expression in other tissues (Supplementary Figure S2). These tissue-specific expression patterns of different SFs potentially contribute to the tissue-specific regulation of AS events in rat.

### Gene coding ability and function prediction

Since current annotations of rat proteins include only 7989 proteins in UniProtKB/Swiss-Prot and 27 902 proteins in UniProtKB/TrEMBL (42), we further predicted the potential coding ability of the newly annotated transcripts in RTR. Of all the 73 074 newly annotated transcripts, 34 213 and 24 728 were considered as highly confident coding transcripts and long noncoding transcripts, respectively (See ‘Materials and Methods’ section and Supplementary Tables S3 and 4). The 34 213 coding transcripts belong to 11 042 genes and these genes all have orthologs in other species. Among the newly annotated transcripts with high confidence in this study, 791 coding transcripts were located in 564 newly annotated genes while 21 301 long noncoding transcripts were located in 17 913 newly annotated genes. Interestingly, 39 newly annotated genes possess both coding and long noncoding transcripts with high confidence, similar to the 231 known genes with this property in Ensembl. Genes such as SRA (43) and VegT (44) have both coding and noncoding transcripts which indicates a gene may possess versatile functions (45). A published RT-PCR experiment confirmed three multi-exon lncRNAs in rat (Supplementary Figure S3D in (28)), two of which (rnor_lincRNA13804051, rnor_lincRNA4244571) are verified as lncRNAs with high confidence in RTR (RTRG.42653.1 and RTRG.13897.2, respectively); another multi-exon lncRNA (rnor_lincRNA4825071) has earlier been added to Ensembl (ENSRNOT00000085828).

**Figure 2.**
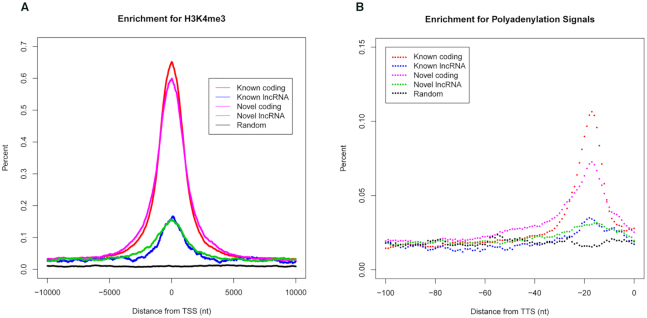
TSS and TTS characterization in RTR. (**A**) ChIP-seq data enrichment for H3K4me3 from male liver at 9 weeks at 10-kb intervals surrounding expressed TSSs (FPKM > 0.5) in male liver at 6 weeks in RTR. (**B**) Hexamer polyA signal (AATAAA, ATTAAA) enrichment in the upstream 100 bp of TTSs in RTR.

To further corroborate active transcription of the newly annotated transcripts with high confidence, we intersected intervals surrounding the TSSs of expressed transcripts in male liver at 6 weeks in RTR with the ChIP-seq open source data for histone 3 lysine 4 trimethylation (H3K4me3) acquired from male liver at 9 weeks in rat (See ‘Materials and Methods’ section and Figure 2A) (28). For comparison, transcripts were classified into four categories: (i) known coding transcripts in Ensembl; (ii) known lncRNAs in Ensembl; (iii) novel coding transcripts with high confidence; and (iv) novel lncRNAs with high confidence. Maximal enrichment of H3K4me3 histone modification at the TSSs of the newly assembled transcripts with high confidence but not at randomly shuffled control regions suggests that these transcripts possess actively regulated promoters.

To support the accuracy of 3′ end of the newly annotated transcripts with high confidence, we intersected intervals in the upstream 100 bp of the transcription termination sites (TTSs) of all newly annotated transcripts with high confidence in RTR with the hexamer polyA signals (AATAAA and ATTAAA) (Figure 2B) (46). Transcripts were classified into four categories for comparison as stated above. Maximal enrichment of the hexamer polyA signals upstream from the TTSs of the newly assembled transcripts with high confidence but not at randomly shuffled control regions suggests that these transcripts possess actively polyadenylation sites.

**Figure 3.**
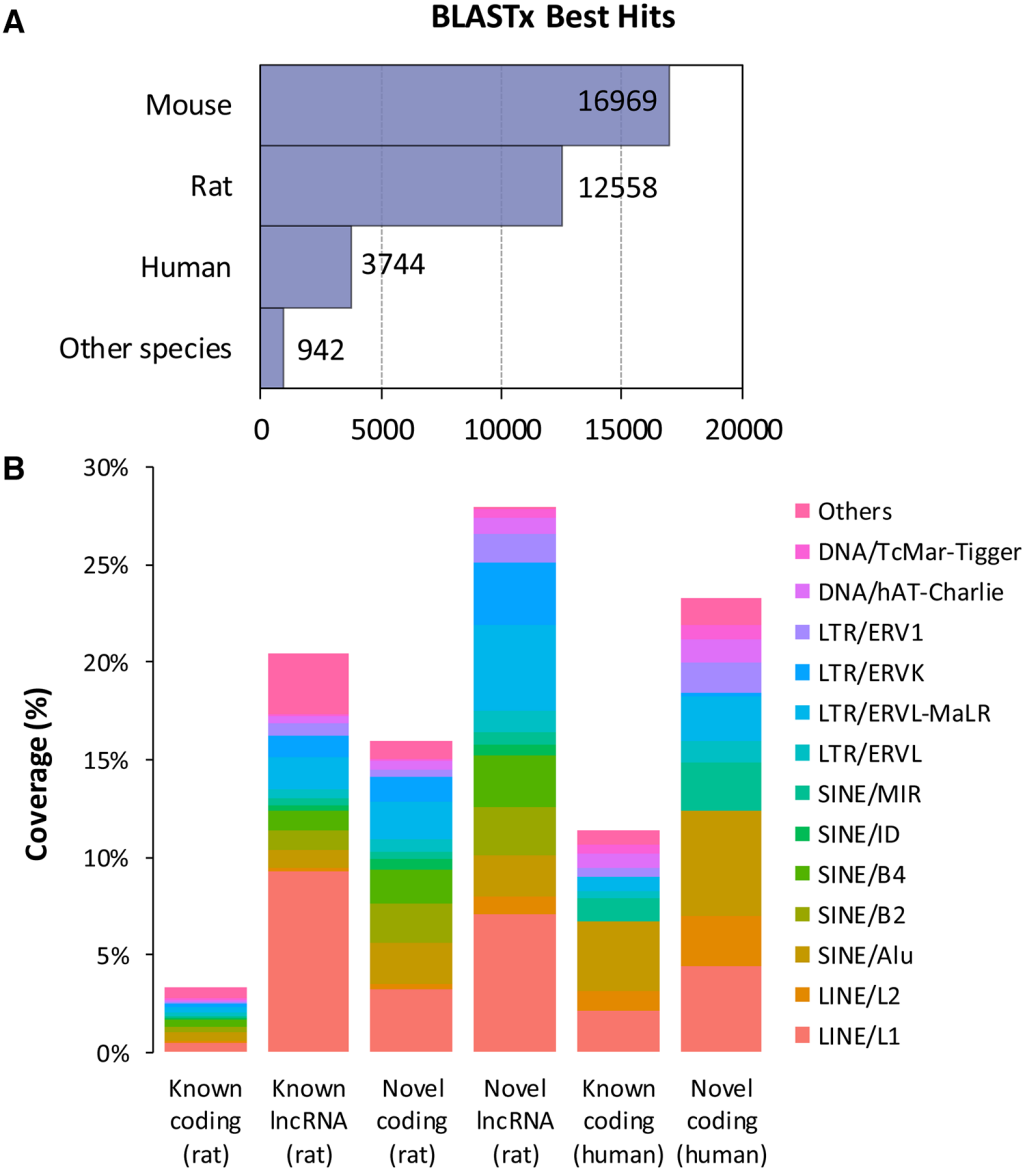
Cross-species feature comparision of transcripts with high confidence in RTR. (**A**) The species distribution of the best BLASTx hits of newly annotated coding transcripts with high confidence in RTR. (**B**) Repetitive content analysis between RTR and human in Ensembl. Sequence-based overlap between exons and TEs was calculated.

The 34 213 newly annotated coding transcripts with high confidence were aligned to known proteins in all organisms in UniProtKB/Swiss-Prot (27) with BLASTx and we only retained the best hits (See ‘Materials and Methods’ section). Because of the relative paucity of rat proteins in the databases used by BLASTx, ∼60% of the best hits were proteins from mouse and human instead of rat (Figure 3A and Supplementary Table S3). This cross-species transcript-protein result supports the premise that the newly annotated transcripts identified in RTR actually code for proteins. There are 81 newly annotated genes encoding newly annotated proteins not appearing in known rat annotations but with unambiguous BLASTx hits in human or mouse (Supplementary Table S5). The novel coding transcripts with high confidence were then annotated with GO terms using Blast2GO suite and 33 589 of them were found associated with GO terms (Supplementary Table S6).

We further checked the cross-species conservation of 34 082 newly annotated junctions in the novel coding transcripts with high confidence; long noncoding RNAs (such as Air and Xist) were not examined because of lack of strong conservation (47). A total of 32 571 of 34 082 junctions belong to genes with homologous genes in human and 32 212 of them belong to the genes with homologous genes in mouse. We then aligned the 32 571 and 32 212 junctions in rat to all the junctions in the corresponding homologous genes in human and mouse, respectively. We found 41.1 and 49.2% of them were conserved in human and mouse, respectively (See ‘Materials and Methods’ section and Supplementary Tables S7 and 8).

### Role of transposable elements in RTR

TEs, also known as repetitive DNA sequences, are spread widely in the genome and are important in genome function and evolution (48). Some of the functional repeat sequences are located in known long noncoding RNAs including Kcnq1ot1 (49) and Xist (50). Figure 3B shows the percentages derived from dividing base-by-base overlap between different types of TEs and exons from different types of transcripts by the total length of transcripts in human and rat. The percentages in both coding and long noncoding newly annotated transcripts with confidence in RTR (15.9 and 28.6%, respectively) are higher than the percentages in the counterparts in Ensembl in rat (3.3 and 20.4%, respectively). The most abundant families in novel transcripts with confidence in RTR are LINE/L1, LINE/L2, SINE/Alu, SINE/B2, SINE/B4, SINE/MIR and LTR/ERVL-MaLR, which is consistent with earlier reports (51–53). To determine whether such increase in RTR is due to the low annotation level of Ensembl in rat, we figured out that repetitive elements accounted for 11.4% of exonic nucleotides of lncRNAs and 23.3% of exonic nucleotides of coding transcripts in Ensembl in human, which are quite close to the counterparts in RTR.

### Expression patterns among different tissues and stages

**Figure 4.**
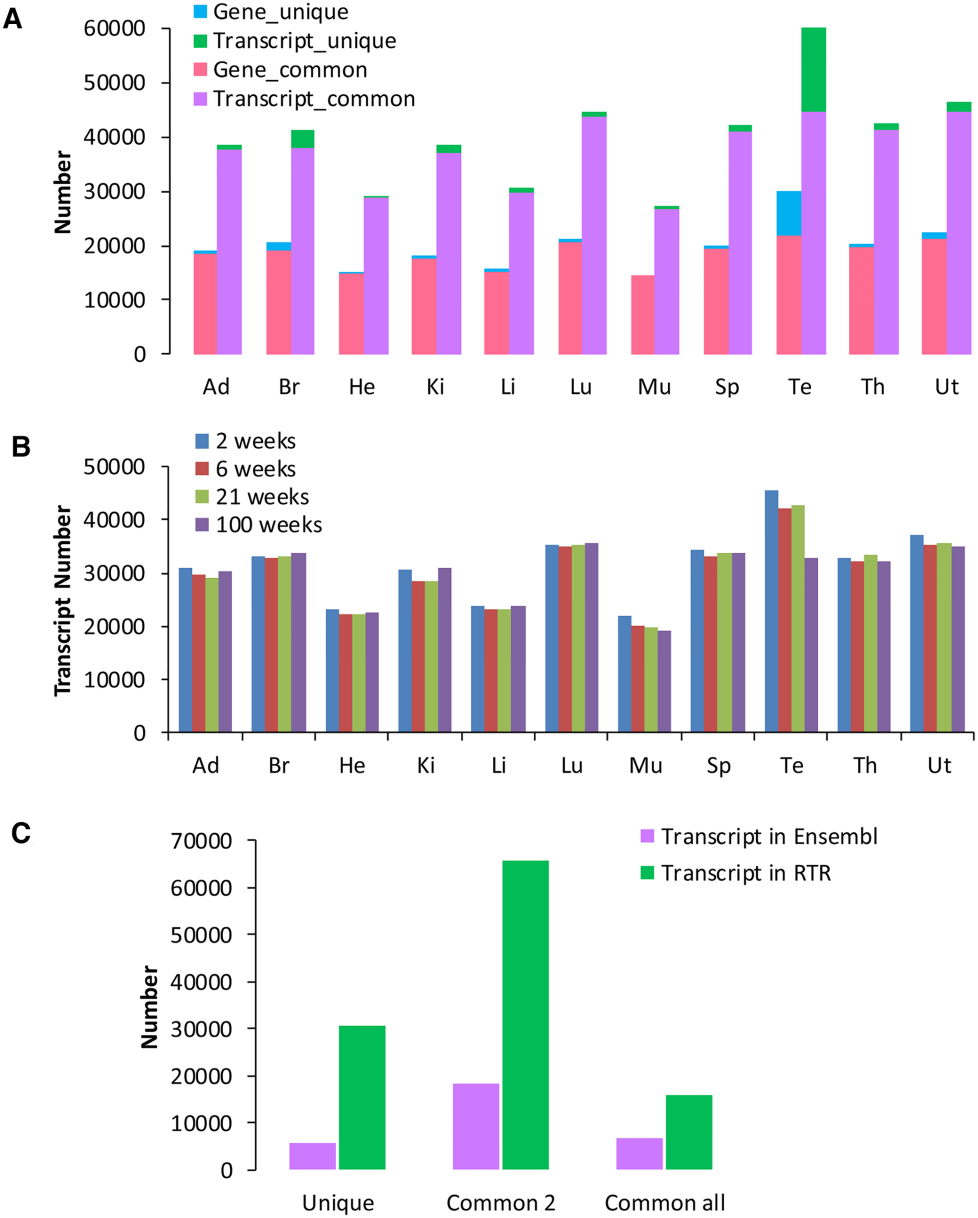
Expression analysis among tissues. (**A**) The distribution of genes and transcripts among tissues. Blue bar means the gene number uniquely expressed in the corresponding tissue. Pink bar means the number of genes not only expressed in the corresponding tissue but also expressed in at least one other tissue. The aggregate length of blue bar and pink bar is the number of genes expressed in the corresponding tissue. The format of gene information shown in the figure is the same as the one of transcript information. Tissues studied are: Ad, adrenal; Br, brain; He, heart; Ki, kidney; Lu, lung; Li, liver; Mu, skeletal muscle; Sp, spleen; Th, thymus; Te, testis; and Ut, uterus. (**B**) The distribution of transcripts under all of the 44 conditions. (**C**) The statistics of transcripts from Ensembl and RTR according to tissues. ‘Common 2’ denotes the transcripts that were identified in at least two tissues. ‘Common all’ denotes the transcripts that were identified in all of the 11 tissues.

We used alignment files (BAM format) acquired from 320 samples to run Stringtie to calculate the expression levels of genes and transcripts in RTR among different tissues and stages in the reference-only mode with the parameter *e* (23). Transcripts/genes which have FPKM > 0.5 in at least two or more samples in a stage and tissue were considered to be expressed in this biological condition. Transcripts/genes which are considered to be expressed in at least one stage in a tissue were considered expressed in this tissue. Expression patterns of different tissues vary considerably. Most of the genes and transcripts detected in one tissue were also expressed in at least one other tissue (designated as ‘common’ in Figure 4A). Testis possesses the most expressed genes and transcripts and the highest proportions of unique genes and transcripts among all organs, which is consistent with what is seen in humans (54). Most of the testis-specific transcripts are involved in spermatogenesis. We also drew a landscape of expression patterns of transcripts in RTR under all of the 44 conditions with different tissues and developmental stages (Figure 4B). We then made a two-way ANOVA analysis on the gene abundance of all genes in RTR and found that tissue type has a significant influence on gene abundance in 44 539 genes while developmental stage has a significant influence in only 25 439 genes (Bonferroni-adjusted, *P*-value < 0.05).

Transcripts in RTR are more tissue-specific than those in existing databases like Ensembl (Figure 4C). Transcripts which are expressed in at least two tissues are much more prevalent than tissue-specific transcripts in both RTR and Ensembl, which demonstrates that there are close associations among tissues. For example, transcripts in heart and muscle are both enriched in several biological processes in Gene Ontology by hypergeometric test (37), e.g. sarcoplasmic reticulum calcium ion transport (GO:0070296), endoplasmic reticulum to cytosol transport (GO:1903513) (Bonferroni-adjusted, *P*-value < 0.05).

There are 15 852 transcripts expressed > 0.5 FPKM among all of the 11 tissues, which we deem as house-keeping transcripts (Supplementary Table S9). These transcripts show enrichment for fundamental functional pathways in KEGG (55) such as metabolic pathways, RNA transport and spliceosome (Bonferroni-adjusted, *P*-value < 0.05). We also found that 11 715 genes are expressed in all these tissues which we deem as house-keeping genes (Supplementary Table S10). A total of 849 house-keeping genes express different isoforms among tissues (Supplementary Table S11) (56).

We then compared the gene abundance of RTR measured by FPKM with an external gene abundance dataset measured by RPKM across rat organ development (E-MTAB-6811) (57). Supplementary Table S12 shows the Spearman's correlation coefficients between the common biological conditions available from the two gene abundance datasets. RTR genes with transcripts in Ensembl were selected to conduct the common gene set for the calculation. Nearly all the coefficients are around 0.85 except for testis at 2 weeks which may result from different reference transcriptomes and technical effects.

### Differences in splicing and gene expression between males and females in adulthood

**Figure 5.**
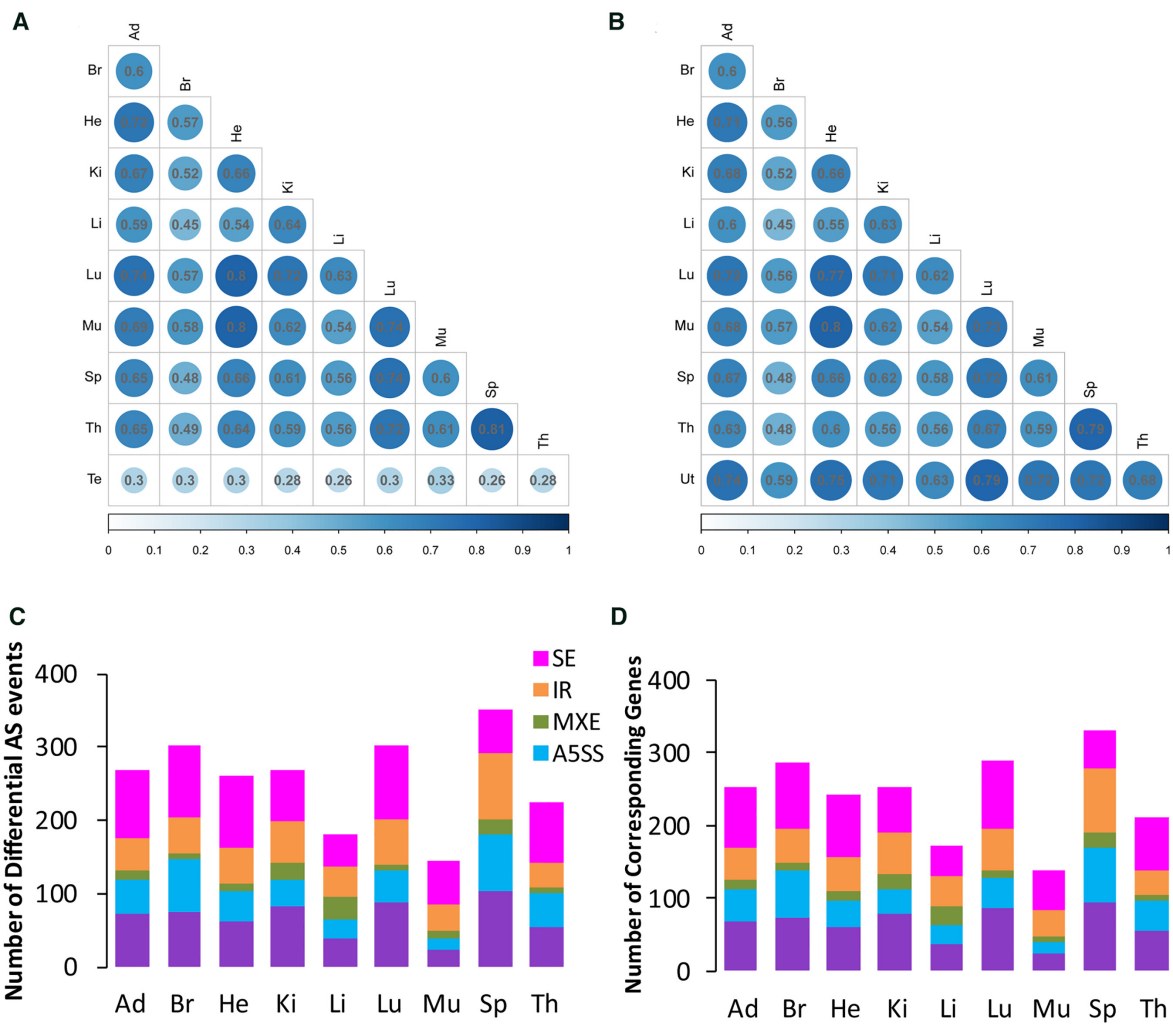
Spearman's correlation coefficients of gene abundance between all organs at 21 weeks in male (**A**) and female (**B**) rats, respectively. Numbers of differential AS events (**C**) and the corresponding genes (**D**) between male and female rats for all nine non-sex organs at 21 weeks.

We calculated the Spearman's correlation coefficients of gene abundance between all organs at 21 weeks in male (Figure 5A) and female rats (Figure 5B), respectively (FDR-adjusted *P*-value < 0.05). We restricted our analysis to the genes which were considered to be expressed in at least one biological condition (FPKM > 0.5) for each comparison. Spleen and thymus, heart and muscle show the maximum values in both sexes as displayed in the hierarchical clustering heatmap of gene expression profiles in Figure 1C in the previous SEQC publication (17). Coefficients between testis and non-sex organs in male rats show the minimum values. Thymus shows the highest sum of absolute value of coefficient difference from all other non-sex organs (0.19), i.e. thymus in females shows lower correlations with all other non-sex organs than the male counterparts, which may result from better thymic function in females compared with males (58).

We then calculated the Spearman's correlation coefficients of gene abundance between male and female rats in different non-sex organs (FDR-adjusted *P*-value < 0.05). Genes were selected as stated above. The coefficients are ∼0.96 except for the coefficient in liver (0.92), which is consistent with the fact that the liver shows the most sex-different DEGs (1478) among non-sex organs as is shown in the result below.

Differential gene expression profiles between male and female rats for all nine non-sex organs at 21 weeks were analyzed with the DESeq2 package and a gene was considered differentially expressed when at least a 1.5-fold difference in expression existed between sexes (|log2FoldChange| > 0.585) plus a FDR-adjusted *P*-value < 0.05 (See ‘Materials and Methods’ section and Supplementary Tables S13 and 14). More genes showed sex-different expression in the liver and kidney as reported in previous publications (17,59). Totally, there are 3044 sex-different DEGs, only 12.5% of which are sex-different DEGs in more than one tissue. More genes were found preferentially expressed in male vs female. Male-dominant genes outnumbered female-dominant genes in each non-sex organ (1951 versus 1212 in all).

We used rMATS to identify five types of AS events between male and female rats for all nine non-sex organs at 21 weeks: SE, IR, MXE, alternative 5′ donor site (A5SS), alternative 3′ acceptor site (A3SS) (36). Among the five major types of differential AS events, SE was the most frequent in most organs except for spleen (See ‘Materials and Methods’ section). In contrast, MXE was the least common differential AS pattern (Figure 5C). Most of the differential spliced genes (DSGs) undergo only one differential AS event (Figure 5C and D).

To assess the global correlation in AS patterns between male and female rats for all nine non-sex organs at 21 weeks, we restricted our analysis to the alternatively spliced cassette exons with at least 20 reads mapped to one of the three exon-exon junctions in an AS event in all samples during comparison. We observed high correlations in the exon inclusion levels of these exons which means high similarities of AS events between male and female rats for all nine non-sex organs (Pearson correlation r: ∼0.98, *P*-value < 2.2e-16) (Supplementary Figure S5). The exon inclusion level of an alternatively spliced cassette exon in any given tissue was estimated from the counts of ESTs mapped uniquely to the exon inclusion or skipping exon–exon junctions.

To analyze DEGs and DSGs between sex gonads, we compared testis and ovary at 16 weeks from the same external rat RNA-seq dataset used above (E-MTAB-6811) with DESeq2 and rMATS after processing the fastq files (35,36). A total of 4013 differential AS events were identified from 2361 DSGs. Similar to the expression trend in non-sex organs, more genes were preferentially expressed in male vs female (9079 versus 5087). Among 2361 DSGs detected in testis vs ovary at 16 weeks, 833 DSGs undergo more than one differential AS event, which is much higher than those multi-differential AS events happened between male and female rats in all nine non-sex organs (Figure 5C and D). Whether a gene is a DEG has no correlation with whether it is a DSG (Chi-square test, *P*-value > 0.05) between male and female rats for the same non-sex organ at 21 weeks and between testis and ovary at 16 weeks.

### RTR presentation in website

RTR is freely available at http://www.unimd.org/rtr/. There are six sections in the website: Home, Browse, Downloads, FAQ, Search and Contact Us. In the Browse section, our website displays all 52 807 genes in the order of their locations in the rat reference genome (rnor6). IDs of newly annotated genes begin with ‘RTRG.’ while IDs of genes/transcripts in known databases (Ensembl, RefSeq) are retained. Transcript information can be searched by clicking the Gene ID field of the queried gene. A gene can be displayed in Genome Browser of UCSC by clicking its Location field. The information in each cell in the table can be displayed in real time by typing key words in the search box. Since there is loading delay in the Browse section owing to the large dataset, we offer the Search section without loading delay. Records can be displayed by typing key words in the search box in the Search section.

For each selected gene, we displayed the expression profile in each tissue and developmental stage in a boxplot. We also calculated the positively/negatively correlated genes of the selected gene by organ. We displayed these highly correlated genes with the selected gene in a table with correlation coefficients from high to low with the minimum value over 0.9 in the organ-specific mode and over 0.7 in the global-organ mode. We only calculated the genes with the expression level over 1 FPKM in at least two samples. By hovering the mouse over the correlated gene in the table, the correlation coefficient is displayed; upon mouse click, the boxplot of this correlated gene is displayed.

## DISCUSSION

RNA-seq data provide great advantages in both biological status and genome coverage for the comprehensive understanding of the transcriptome. By developing and applying novel software and streamlined analysis pipelines for integrating dispersed RNA-seq data into reliable and reusable data resources, transcriptome analysis and mining to understand biological function can be enhanced. In this study, we reconstructed a rat transcriptome by combining existing rat annotation resources with rat BodyMap data. This new comprehensive rat transcriptome now has completeness and annotation comparable to the well-annotated mouse transcriptome. Transcripts in RTR cover over 97% of the identified transcripts in 5 other datasets which verifies the accuracy of RTR (Supplementary Table S1). This small difference may be attributable to unique transcripts in a certain individual and sequencing errors.

Although the analysis pipeline has been documented, reliable sample-specific transcriptome needs improvement in each link. Since novel coding transcripts of high confidence in RTR were predicted in-silico, it is worthwhile to consider some additional studies to produce specific antibodies of some interesting newly discovered coding isoform sequences of genes. As the transcriptome is better defined, the accurate definition of each gene becomes more complicated. RTR is a re-annotated transcriptome derived from common tissues of different developmental stages under normal experimental conditions. In its present development, RTR may not contain all genes/transcripts because RNA-seq data are not available from every rat tissue/cell type under all possible experimental conditions, diseases and pathologies. Although expression of genes/transcripts may oscillate with a period of about 24 h (60), the RTR website only covers the expression of genes/transcripts at one point in the 44 conditions.

However, RTR appears to accurately and comprehensively represent the transcriptome landscape of the rat to a considerable extent (Supplementary Table S15). For example, RTR contained 98.5% of transcripts identified in a normal liver sample (SRP028932; Supplementary Table S1), while only 94.3% were identified in hypertensive liver samples from the same experiment. Further, RTR identified 98.6% of transcripts in an independent study of the whole kidney (SRP041131; Supplementary Table S1) but only 97.2% of the transcripts in the S1 renal proximal tubule from the same experiment. Thus, for a more complete definition of the rat transcriptome, additional RNA-seq data is required from tissues and cell types not represented in the Rat BodyMap database, as well as transcripts from rats with disease/pathological states and under different environmental conditions.

The rat has been widely used in toxicological studies. However, results from preclinical findings in the rat model sometimes cannot be reliably extrapolated to human clinical trials. The underlying mechanisms behind this problem have not been fully understood. The incomplete annotation for the rat genome and transcriptome has seriously inhibited the cross-species comparison studies necessary to investigate these inconsistencies between rat and human, and this may have contributed to the limitations of this animal model. Our new comprehensively re-annotated transcriptome provides a valuable reference for biomedical research in understanding human diseases, and performing studies on drug toxicity and adverse effects using the rat animal model.

Of the detected 73 074 newly annotated transcripts, 19.3% of the transcripts could not be assigned a coding function by our pipeline. Inconsistent results from software used for the judgement of coding ability appeared to be the cause. Newly annotated transcripts with ambiguous coding ability may be due to shortcomings of these softwares or because a transcript may act in both coding and noncoding roles (45,61).

This comprehensive re-analysis of the rat BodyMap RNA-seq data significantly expands the rat transcriptome. The numbers of genes and transcripts were increased greatly over existing databases. The ratio of multi-isoform genes has been extensively increased from 15.4% in Ensembl to 34.3% in RTR (Figure 1). Based on the information by tissue in RTR, we found that most of the transcripts are widely shared among tissues and we also identified a more comprehensive set of house-keeping genes and transcripts than ever before. This new rat transcriptome provides an essential reference for genetics and gene expression studies in rat disease and toxicity models.

## Supplementary Material

gkaa638_Supplemental_Files
