# Supplementary material for: A comprehensive rat transcriptome built from large scale RNA-seq-based annotation
Source: Nucleic Acids Res. 2020 Aug 4;48(15):8320–31. doi: 10.1093/nar/gkaa638 (PMC7470976; doi:10.1093/nar/gkaa638)
Supplement: gkaa638_Supplemental_Files [file gkaa638_supplemental_files.zip › Supplementary Figures and Table Legends.docx]

**Supplementary Figures**

**Figure S1.** The distribution of alternative splicing events occurring in Rat Ensembl release (version 97).


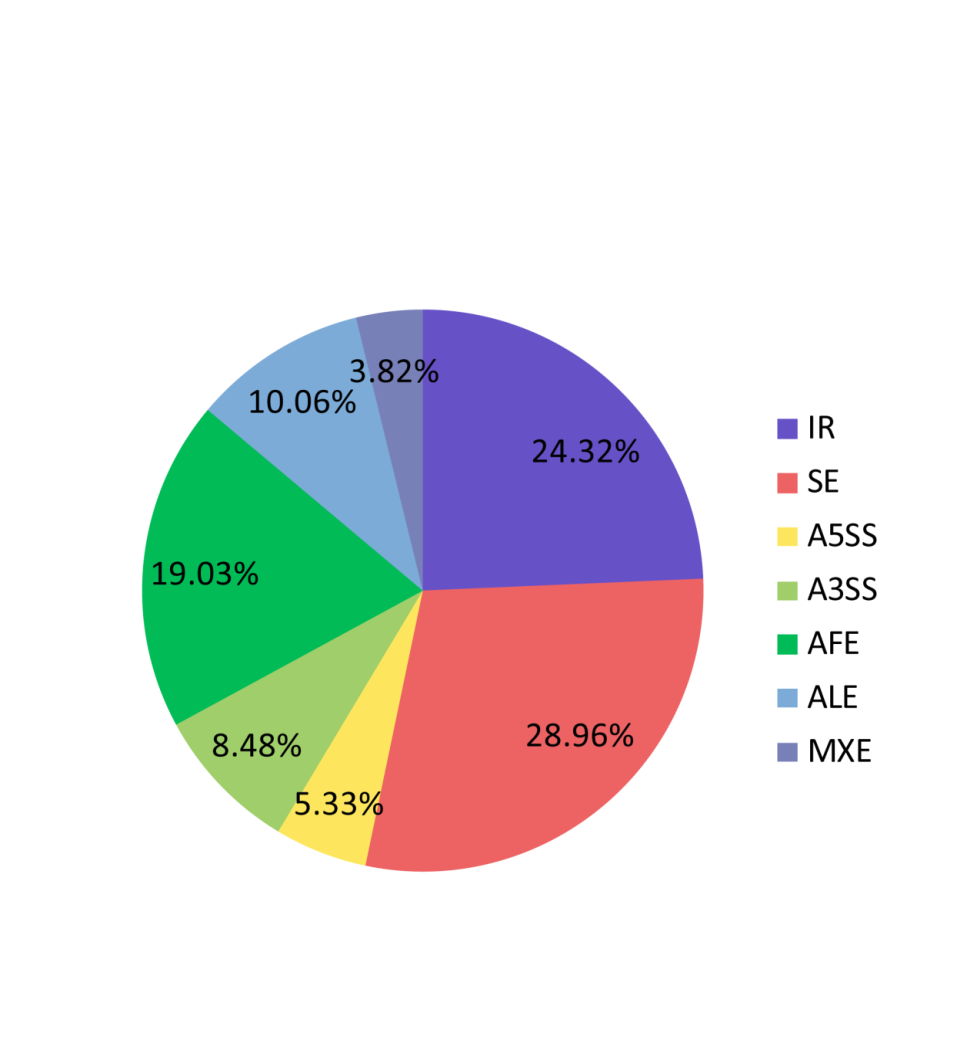


**Figure S2.** Tissue-specific expression pattern of splicing factors. The trailing numbers of x-axis labels refer to animal age in weeks. Tissues studied are: Ad, adrenal; Br, brain; He, heart; Ki, kidney; Lu, lung; Li, liver; Mu, skeletal muscle; Sp, spleen; Th, thymus; Te, testis; and Ut, uterus.


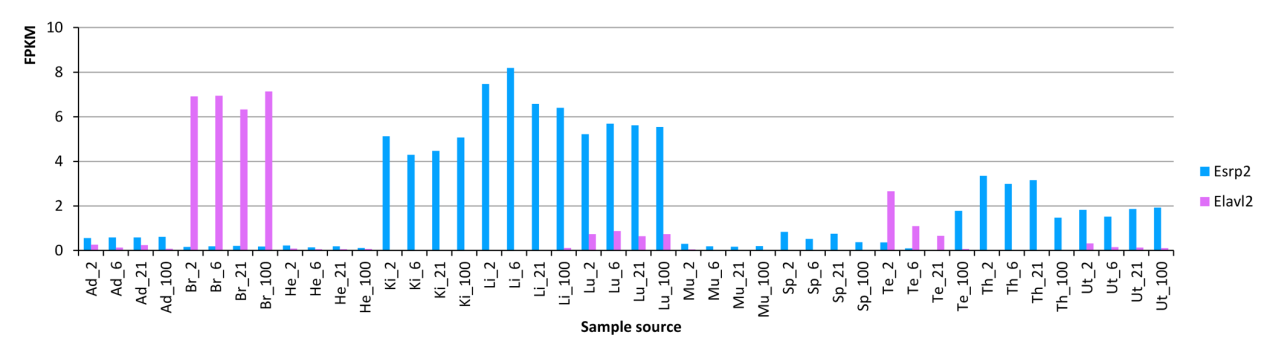


**Figure S3** The pipeline to generate RTR.

**
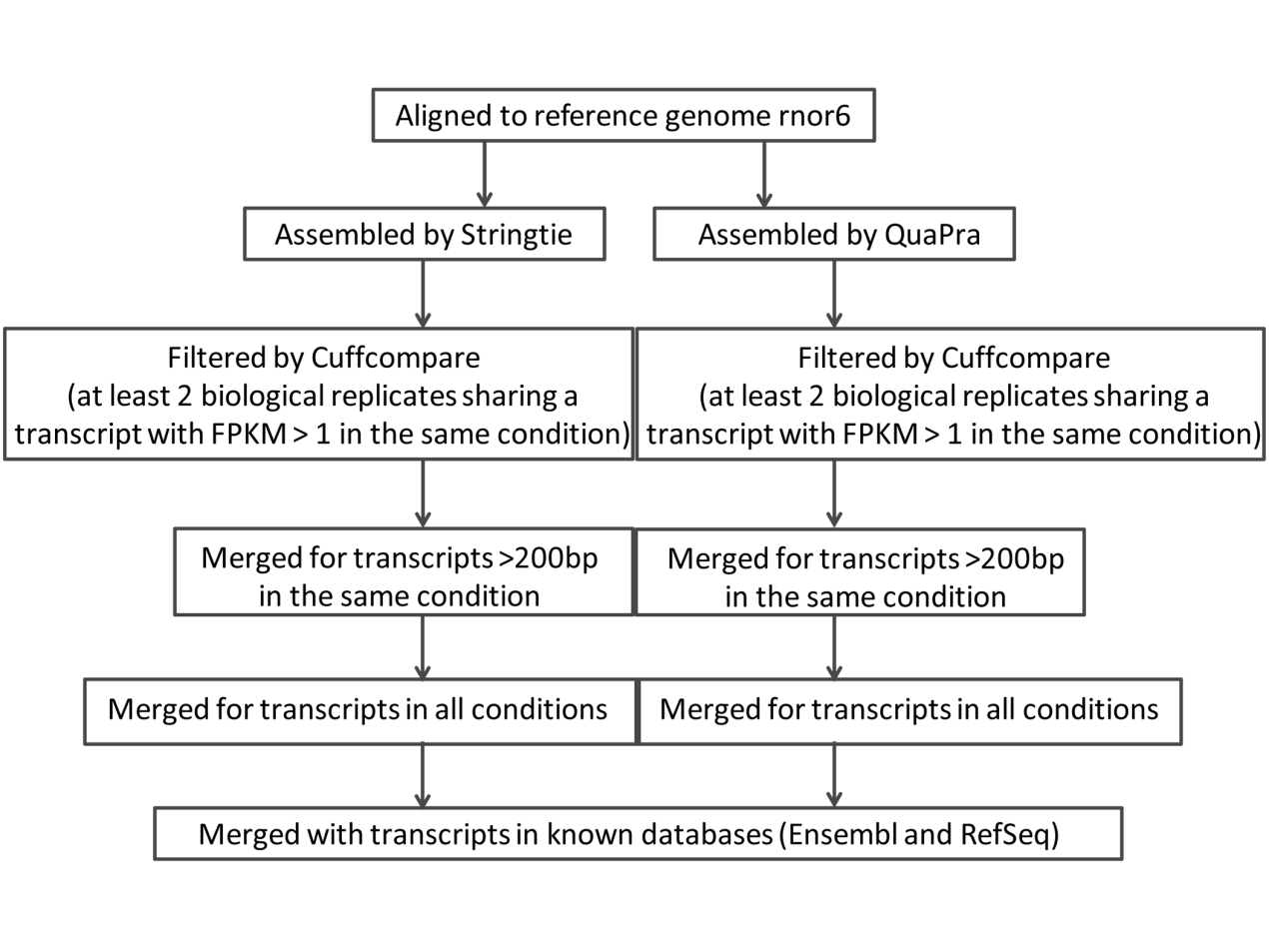
**

**Figure S4** (A) Comparison of the numbers of genes and transcripts in different transcriptomes. QuaPra covers most of the transcripts detected by Stringtie. (B) Uncovered annotated gene number when using Stringtie and QuaPra. Stringtie and QuaPra were not able to recall 8305 and 5238 genes longer than 200bp in known databases, respectively. There are 4485 genes in known databases which cannot be covered by both of the assemblers.

**
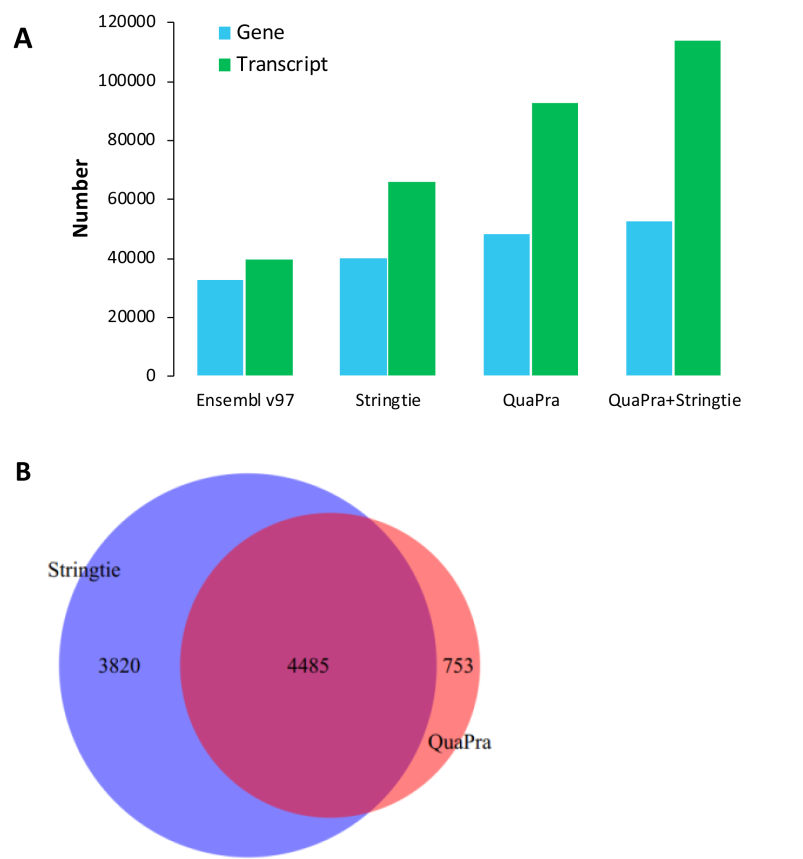
**

**Figure S5** The scatter plots of the exon inclusion levels in male and female groups of biological replicates from non-sex organs at weeks. The two red lines define the area where  |ΔPercent spliced in (PSI)| < 0.1. ΔPercent spliced in (PSI) = IncLevel 1 – IncLevel 2; IncLevel: exon inclusion level.


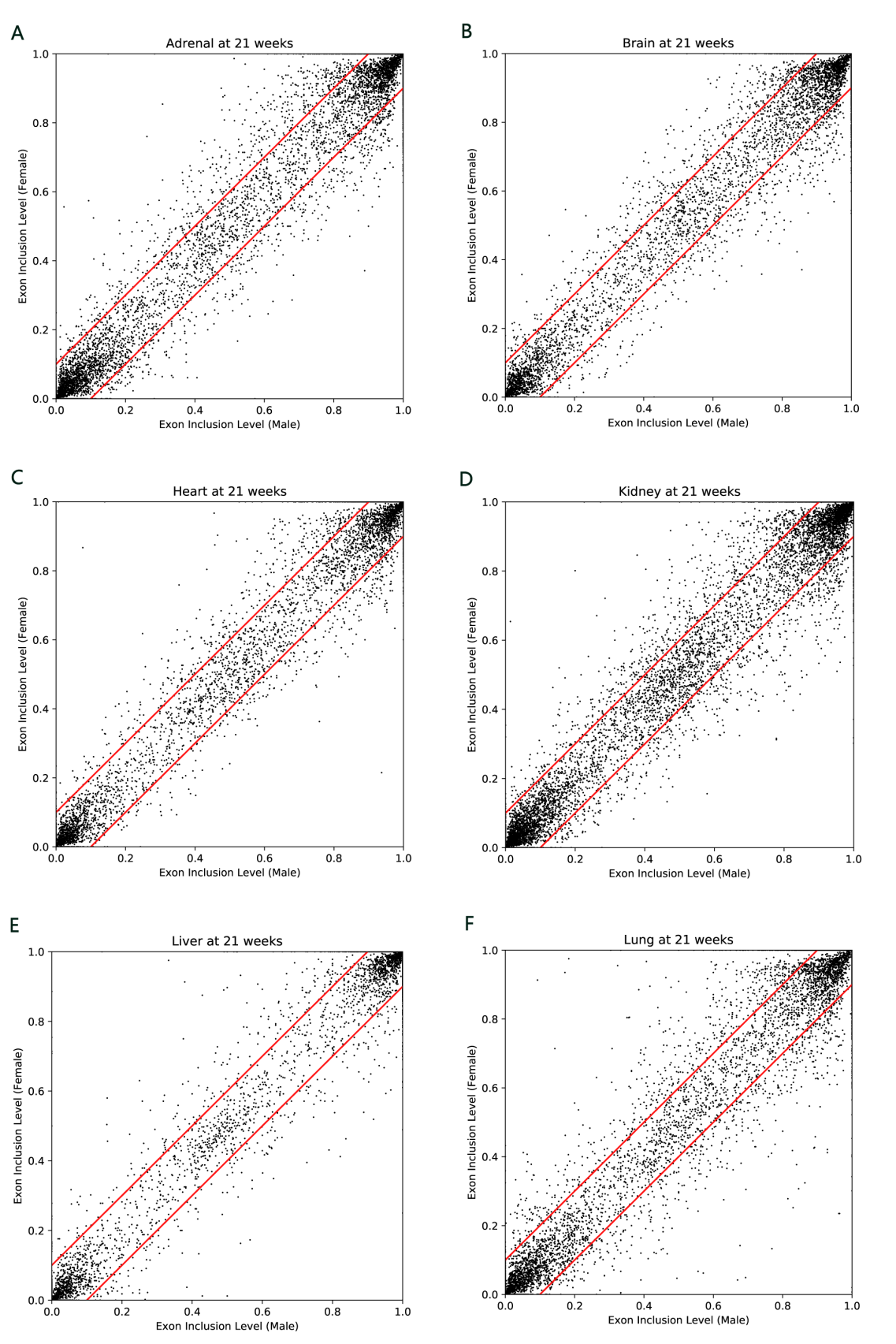


**Supplementary Table Legends**

**Table S1.** Precision of RTR in 5 datasets derived from the same organs in RTR.

**Table S2.** Splicing factors in RTR.

**Table S3.** High confident novel coding transcripts in RTR.

**Table S4.** High confident novel lncRNAs in RTR.

**Table S5.** Newly annotated genes encoding newly annotated proteins not appearing in known rat annotations but with unambiguous BLASTx hits in human or mouse.

**Table S6.** Novel coding transcripts with high confidence in RTR and the associated GO terms annotated with Blast2GO suite.

**Table S7.** Novel splice junctions in coding transcripts with high confidence in RTR conserved in human. The coordinates are shown in 0-based format.

**Table S8.** Novel splice junctions in coding transcripts with high confidence in RTR conserved in mouse. The coordinates are shown in 0-based format.

**Table S9.** House-keeping transcripts in RTR.

**Table S10.** House-keeping genes in RTR.

**Table S11.** House-keeping genes with different isoforms among tissues.

**Table S12.** Spearman correlation coefficients between the gene abundance of RTR measured by FPKM with an external gene abundance dataset measured by RPKM.

**Table S13.** Numbers of sex-specific DEGs for all nine non-sex organs at 21 weeks.

**Table S14.** Sex-specific DEGs for all nine non-sex organs at 21 weeks.

**Table S15.** Precision of RTR in 5 datasets derived from different organs or experimental conditions from RTR.
